# TM9SF4 is a novel regulator in lineage commitment of bone marrow mesenchymal stem cells to either osteoblasts or adipocytes

**DOI:** 10.1186/s13287-021-02636-8

**Published:** 2021-11-13

**Authors:** Libo Yu, Mingxu Xie, Fengjie Zhang, Chao Wan, Xiaoqiang Yao

**Affiliations:** 1 School of Biomedical Sciences, Heart and Vascular Institute and Li Ka Shing Institute of Health Science, Faculty of Medicine, The Chinese University of Hong Kong, Shatin, Hong Kong SAR People’s Republic of China; 2 Shenzhen Institute of Translational Medicine, Shenzhen Second People’s Hospital, The First Affiliated Hospital of Shenzhen University, Shenzhen, People’s Republic of China; 3 MOE Key Laboratory for Regenerative Medicine, School of Biomedical Sciences, Faculty of Medicine, The Chinese University of Hong Kong, Hong Kong, People’s Republic of China; 4 Centre for Cell and Developmental Biology, State Key Laboratory of Agrobiotechnology, School of Life Sciences, The Chinese University of Hong Kong, Hong Kong, People’s Republic of China

**Keywords:** TM9SF4, MSCs, Osteoblasts, Adipocytes, Osteoporosis

## Abstract

**Background:**

Osteoporosis is a common bone disease in elderly population caused by imbalanced bone formation and bone resorption. Mesenchymal stem cells (MSCs) are responsible for maintaining this bone homeostasis. The phenotype of transmembrane 9 superfamily 4 (TM9SF4) knockout mice suggests a relationship between TM9SF4 proteins and bone homeostasis. But the effect of TM9SF4 in osteology has never been reported. In the present study, we investigated the function of TM9SF4 in MSC differentiation commitment, as well as its role in osteoporosis.

**Methods:**

Primary bone marrow MSCs, isolated from TM9SF4 wildtype (TM9SF4^+/+^) and knockout (TM9SF4^−/−^) mice, were induced to differentiate into osteoblasts or adipocytes, respectively. The osteogenesis was examined by qRT-PCR detection of osteogenic markers, ALP staining and Alizarin Red S staining. The adipogenesis was tested by qRT-PCR quantification of adipogenic markers and Oil Red O staining. The cytoskeletal organization of MSCs was observed under confocal microscope. The osteoporotic model was induced by ovariectomy in TM9SF4^+/+^ and TM9SF4^−/−^ mice, followed by Toluidine blue and H&E staining to assess lipid accumulation in trabecular bones, as well as micro-computed tomography scanning and immunohistochemistry staining for bone mass density assessment. The experiments on signaling pathways were conducted using qRT-PCR, Western blot and Alizarin Red S staining.

**Results:**

We determined the role of TM9SF4 in MSC differentiation and found that TM9SF4^−/−^ MSCs had higher potential to differentiate into osteoblasts and lower capability into adipocytes, without affecting osteoclastogenesis in vitro. In ovariectomy-induced osteoporotic model, TM9SF4^−/−^ mice retained higher bone mass and less lipid accumulation in trabecular bones, indicating an important role of TM9SF4 in the regulation of osteoporosis. Mechanistically, TM9SF4-depleted cells showed elongated actin fibers, which may act through mTORC2/Akt/β-catenin pathway to promote their commitment into osteoblasts. Furthermore, TM9SF4-depleted cells showed higher activity of canonical Wnt pathway, suggesting the participation of Wnt/β-catenin during TM9SF4-regulated osteogenesis.

**Conclusions:**

Our study demonstrates TM9SF4 as a novel regulator for MSC lineage commitment. Depletion of TM9SF4 preferentially drives MSCs into osteoblasts instead of adipocytes. Furthermore, TM9SF4^−/−^ mice show delayed bone loss and reduced lipid accumulation during ovariectomy-induced osteoporosis. Our results indicate TM9SF4 as a promising target for the future clinical osteoporotic treatment.

**Supplementary Information:**

The online version contains supplementary material available at 10.1186/s13287-021-02636-8.

## Introduction

Osteoporosis is a skeletal disease characterized by decreased bone density and increased fracture risk. Osteoporosis has become a major health problem in aging population. The National Osteoporosis Foundation has reported that up to one in three women and one in five men aged over 50 undergo osteoporotic fractures.

Mesenchymal stem cells (MSCs) residing in the bone marrow play a crucial role in the maintenance of bone stability. Bone marrow MSCs are capable of self-renewing and differentiating into multiple cell lineages, including osteoblasts, adipocytes and chondrocytes [1, 2]. As the common progenitor cells of osteoblasts and adipocytes, MSCs are delicately balanced in their differentiation commitment. Their differentiation selection between osteogenesis and adipogenesis is often competing and reciprocal [3]. During osteoporosis, MSCs exhibit a reduced capacity to differentiate into osteoblasts and an increased capacity into adipocytes, which results in a reduction in bone formation and an increase in marrow fat accumulation [4, 5]. Such a shift of MSC differentiation into adipocytes rather than osteoblasts contributes to osteoporosis [2, 4].

Several signaling pathways, including Wnt/β-catenin, BMP and Notch pathways, play important roles in the differentiation of MSCs toward osteoblasts or adipocytes [6–8]. Among them, canonical Wnt/β-catenin pathway is the most effective and influential one [2]. Specifically, binding of Wnt ligands to cell surface receptors prevents the phosphorylation and degradation of β-catenin. The un-phosphorylated β-catenin molecules are then translocated into the nucleus to stimulate the expression of osteogenesis-related genes and, at the same time, to inhibit the expression of adipogenic genes [9–11].

In addition to chemical signals, the differentiation of MSCs can also be regulated by physical signals such as cell shape and cytoskeleton alteration [12]. Indeed, actin polymerization was found to promote MSC differentiation into osteoblasts, whereas actin depolymerization favors MSC commitment to adipocytes [12–15]. It is believed that cytoskeleton and F-actin polymerization act through mTORC2/Akt/β-catenin signaling cascade to promote MSC differentiation toward osteogenic lineage but disfavor its differentiation into adipogenic lineage commitment [16–18].

However, despite the above-mentioned advance in knowledge about MSC differentiation, in general, the molecular mechanisms regulating the switch between osteoblasts and adipocytes are still not well defined.

TM9SF4 belongs to the transmembrane 9 superfamily (also known as TM9SF or nonaspanins) [19]. Functional studies show that TM9SF4 participates in cell adhesion, phagocytosis, autophagy and cancer chemoresistance [20–24]. Interestingly, TM9SF4 knockout mice show abnormal thoracic cage shape and vertebrate transformation, suggesting a possible role of TM9SF4 in bone formation/development (https://www.mousephenotype.org/data/genes/MGI:2139220#phenotypesTab). In addition, in *Drosophila* macrophage, TM9SF4 mutant displays altered cell shape and defective actin organization [21], implicating an involvement of TM9SF4 in cytoskeleton remodeling.

In the present study, we set about exploring the role of TM9SF4 in regulating MSC differentiation. Our results demonstrated that TM9SF4 proteins regulated the switch between osteogenic and adipogenic differentiation of MSCs. Knockout of TM9SF4 promoted osteogenic differentiation but suppressed adipogenic differentiation of MSCs. In addition, knockout of TM9SF4 reduced osteoporosis in ovariectomized mouse model. The underlying signaling cascades might involve Wnt//β-catenin and F-actin/mTORC2/Akt/β-catenin.

## Materials and methods

### Isolation and characterization of primary murine mesenchymal stem cells from bone marrow

TM9SF4^+/+^ and TM9SF4^−/−^ mice were obtained as described before [23]. Female or male mice at the age of 6–8 weeks were euthanatized by CO_2_. Femurs and tibias were dissected out and cut into small pieces in ice-cold αMEM, followed by vortex for 1 min to release bone marrow cells into the medium. The medium was firstly filtered through a 70-μm membrane to remove bone spicules and cell clumps, followed by centrifugation at the speed of 1500 g for 5 min at 4 °C. The cell pellet was resuspended with αMEM (10% FBS, 100 units/mL penicillin and 100 units/mL streptomycin) and cultured in a humidified incubator with 5% CO_2_ at 37 °C. The medium was changed every day from the second day until there were no floating cells. After that, the medium was changed every 3 days until the cells were fully confluent in the culture dishes. The cells were dislodged with 0.25% trypsin/1 mM EDTA at 37 °C for 2 min. After passage, the cells were continuously cultured in αMEM (10% FBS, 100 units/mL penicillin and 100 units/mL streptomycin) until use.

The phenotype of primary MSCs was verified after at least three passages. Specifically, MSCs from the bone marrow of TM9SF4^+/+^ and TM9SF4^−/−^ mice were firstly incubated with primary antibodies, including three MSC-positive markers (CD29, CD44, CD105, all from R&D Systems) and two MSC-negative markers (CD45 and CD11b, all from R&D Systems), followed by stained with fluorescent secondary antibody (Invitrogen). Flow cytometric experiments were performed using BD Fortessa Cell Analyzer and analyzed by FlowJo 10.4.

### Osteogenic and adipogenic differentiation

For osteogenic differentiation, MSCs or MC3T3-E1 cells were cultured in osteogenic medium (αMEM with 10% FBS, 100 nM dexamethasone, 50 μM ascorbic acid, 20 mM β-glycerol phosphate, 100 units/mL penicillin and 100 units/mL streptomycin) for specific time depending on the needs of experiments. For adipogenic differentiation, MSCs were cultured in adipogenic medium (HG-DMEM with 10% FBS, 500 nM dexamethasone, 50 μM indomethacin, 500 μM isobutyl methylxanthine, 10 μg/mL insulin, 100 units/mL penicillin and 100 units/mL streptomycin) for 5 or 7 days.

### Osteoclast differentiation

Bone marrow-derived monocytes were used for osteoclast differentiation in vitro. The basic medium for monocytes was RPMI1640 (10% FBS, 20 ng/mL macrophage colony stimulating factor (M-CSF), 100 units/mL penicillin and 100 units/mL streptomycin). Three days after isolation, monocytes could fully adhere to the culture plates. The medium was changed to RPMI1640 with 20 ng/mL M-CSF every 3 days and continuously cultured for another 5 days. After that, the inducing medium was changed to RPMI1640 with 20 ng/mL M-CSF and 20 ng/mL receptor activator for nuclear factor-κB Ligand (RANKL). Then, the medium was changed every 3–4 days for a total induction period of 2 weeks.

### Cell staining

For ALP staining, the cells were fixed with 4% PFA for 15 min at room temperature, followed by incubated with 1-Step NBT-BCIP (ThermoFisher Scientific) at room temperature for 5–15 min until the desired color developed.

For Alizarin Red S staining, the cells were fixed with 4% PFA for 15 min at room temperature, then incubated with 0.2% Alizarin Red S (Sigma) in the dark for 10 min at room temperature and rinsed in distilled water.

For Oil Red O staining, cells were fixed with 4% PFA. Then, the cells were rinsed with 60% isopropanol for 2 min, followed by stained with freshly prepared Oil Red O working solution for 15 min at room temperature. The cells were then rinsed with 60% isopropanol for 30 s. The nuclei were lightly stained with alum haematoxylin and rinsed in distilled water.

For TRAP staining, the cells were fixed with 4% PFA for 15 min at room temperature, followed by stained with Sigma Diagnostics™ Acid Phosphatase Kit (ThermoFisher Scientific) according to the manufacturer’s instruction. The activity of tartrate-resistant acid phosphatase (TRAP) was also detected by TRAP Assay Kit (Beyotime) after cell lysis.

### siRNA transfection and lentivirus infection

Primary MSCs or MC3T3-E1 cells were seeded on 6-well plates to reach 50–60% confluence at the time of transfection. In total, 5 μL siRNA-mate (GenePharma) and 10 μL siRNA-cocktail (10 mM, GenePharma) were added to the Opti-MEM, followed by vortex for 10 s. After 20-min incubation at room temperature, the mixture was added to the medium for continuous culture.

As for construction of shRNA-loaded lentivirus, HEK-293FT cells were plated on 10-cm dishes to reach 50–60% confluence. pMD2.G, psPAX2 and target plasmids (pLKO.1-puro-shTM9SF4 or pLKO.1-puro-shScramble) were mixed with Lipofectamine™ 3000 Reagent and P3000™ Reagent, diluted in Opti-MEM, and incubated for 20 min. The mixture was then added to HEK-293FT cells for continuous culture. The supernatants were harvested at 48 h and 72 h post-transfection, followed by cell debris removal. To transduce cells with lentivirus, MC3T3-E1 cells were seeded on 6-well plates and inoculated with 2 mL/well lentivirus-containing medium for 12 h. Then, the cells were fed with fresh medium. Two days after inoculation, the cells were selected with specific antibiotics and maintained for at least 1 week before further use.

### Immunofluorescence staining

After fixation, the cells were permeabilized with 0.2% Triton X-100 for 10 min and blocked with 5% BSA for 30 min at room temperature. Then, the cells were incubated with primary antibody at 4 °C overnight, followed by secondary antibody (1:1000) incubation for 1 h and DAPI staining for 10 min if necessary. After rinsing, the cells were imaged with SP8 confocal system.

### qRT-PCR

Total RNA was isolated with RNAiso Plus (Takara). The complementary DNA was generated from 1–2 μg total RNA with High-Capacity cDNA Reverse Transcription Kit (Thermo Fisher Scientific). mRNAs were quantified using specific primers with SYBR Green PCR Master Mix (Takara). The sequence of primers is shown in Table 1.

**Table 1:** Primer list

Name	Sequence
*Acp5*	F: GCAGTATCTTCAGGACGAGAAC; R: TCCATAGTGAAACCGCAAGTAG
*Adn*	F: CATGCTCGGCCCTACATGG; R: CACAGAGTCGTCA TCCGTCAC
*Alp*	F: AACCCAGACACAAGCATTCC; R: GCCTTTGAGGTTTTTGGTCA
*Bmp2*	F: ATCTGTACCGCAGGCACTCA; R: GGCCGTTTTCCCACTCATCT
*Cebpb*	F: GCAAGAGCCGCGACAAG; R: GGCTCGGGCAGCTGCTT
*Ctsk*	F: CTTAGTCTTCCGCTCACAGTAG; R: ACTTGAACACCCACATCCTG
*Fabp4*	F: TCACCATCACCTATGGACCCA; R: TCCAGTTCGCACTCCTCCC
*Gapdh*	F: AGGTCGGTGTGAACGGATTTG; R: TGTAGACCATGTAGTTGAGGTCA
*Nfatc1*	F: CTCGAAAGACAGCACTGGAGCAT; R: CGGCTGCCTTCCGTCTCATAG
*Ocn*	F: CCTGAGTCTGACAAAGCCTTCA; R: GCCGGAGTCTGTTCACTACCTT
*Opg*	F: ACCCAGAAACTGGTCATCAGC; R: CTGCAATACACACACTCATCACT
*Opn*	F: CCCGGTGAAAGTGACTGATT; R: TTCTTCAGAGGACACAGCATTC
*Osx*	F: CCTCTCGACCCGACTGCAGATC; R: AGCTGCAAGCTCTCTGTAACCATGAC
*Pparg*	F: TTGATTTCTCCAGCATTTC; R: TGATCGCACTTTGGTATT
*Runx2*	F: GAATGGCAGCACGCTATTAAATCC; R: GCCGCTAGAATTCAAAACAGTTGG
*Src*	F: ATGTGGAGCGGATGAACTATG; R: GGCTGTGTATTCGTTGTCTTC
*Tm9sf4*	F: GGAGTCGCGCCAATCAATTTC; R: GGCAGAAGGGCAATGAGTAGT
*Wnt1*	F: GGTTTCTACTACGTTGCTACTGG; R: GGAATCCGTCAACAGGTTCGT
*Wnt2*	F: TCCCAGATTCCAACAACCCAG; R: AACTGATGGGACAGTGAGGAA
*Wnt3*	F: TGTGTCCAAGCTGCCTCTAC; R: GAACAACAGAAAGGGGCGTG
*Wnt8a*	F: GCCTATCTGACCTACACCGC; R: GCTCTGGCATCCTTCCCTTT
*Wnt8b*	F: GGTATCTATCCCTCCCGCCT; R: TCCAGCATTGAGCGACCATT
*Wnt10a*	F: TTGACATTCCTCCGCTCACC; R: TAGTTTTCTTCCCCGGTGCC
*Wnt10b*	F: CCCTCCCTTTTACCCTCCCT; R: CGGGAAGTTTAAGGCCCAGA

### Western blot

Total protein was extracted with RIPA (Beyotime). 20–40 μg protein was loaded to sodium dodecyl sulfate polyacrylamide gel and separated at 80 V for 2 h. After electrophoresis, the protein on the gel was electro-transferred onto PVDF membrane at 100 V for 90 min. Non-specific binding sites were blocked with 5% BSA for 1 h at room temperature. The membrane was incubated with the primary antibody at 4 °C overnight, followed by secondary antibody incubation for 1 h at room temperature. The antibodies are listed in Table 2.

**Table 2:** Antibody list

Name	Supplier	Identifier
ActinGreen™ 488 ReadyProbes	Invitrogen	Cat# R37110
Alexa Fluor 546 goat anti-rabbit IgG	Invitrogen	Cat# A11035
Alexa Fluor 546 goat anti-mouse IgG	Invitrogen	Cat# A11003
Anti-CD11b	R&D Systems	Cat# SC018
Anti-CD105		
Anti-CD29		
Anti-CD44		
Anti-CD45		
Anti-β-actin	Proteintech	Cat# 20536-1-AP
Anti-TM9SF4	Proteintech	Cat# 25595-1-AP
Anti-β-catenin	Proteintech	Cat# 51067-2-AP
Anti-GAPDH	Proteintech	Cat# 60004-1-Ig
Anti-V5	Invitrogen	Cat# R960-25
Anti-pAkt	Cell Signaling Technology	Cat# 9271
Anti-Rictor	Biorbyt	Cat# orb536100
Anti-mTOR	Cell Signaling Technology	Cat# 2983
Anti-Col1a1	Abcam	Cat# ab34710

### Ovariectomy

The female mouse (12 weeks) was laid on its ventral surface after being anaesthetized with 80 mg/kg ketamine and 5 mg/kg xylazine. A small midline dorsal skin incision was made approximately halfway between the middle of the back and the base of the tail. The ovaries were pulled out through the muscle incision by grasping the periovarian fat. The junction between the fallopian tube and the uterine horn, together with all accompanying blood vessels and fat, was severed with a single cut. Then, the uterine horn was returned into the abdominal cavity. The wound was dressed after ovariectomy. The mice were kept feeding with normal diet for 3 months after surgery.

### Micro-computed tomography

Femurs of mice were fixed with 4% PFA at 4 °C for 5 days. The tissues were transferred to 75% ethanol before micro-computed tomography (micro-CT) scanning. The scanning range covered about 8 mm above the extremitas inferior of femurs. The energy for scanning is 70 kV. The threshold for analysis is 220–1000. Four hundred pictures from the end of growth plates were selected for data reconstruction and analysis.

### Immunostaining

The tibias of mice were fixed with 4% PFA at 4 °C for 5 days, followed by being steeped in 10% EDTA at 37 °C for 2 weeks. After that, the tissues were dehydrated and embedded for microtomy. The embedded sections were dewaxed before staining.

For H&E staining, the sections were incubated with Mayer’s hematoxylin for 1–2 min until the desired color developed. After washing, the slides were dipped quickly in 0.1% acid alcohol and Scott’s Tap Water Solution successively for bluing. The cytosol was then stained with eosin for 1 min. After washing, the sections were dehydrated and mounted.

For immunohistochemistry (IHC) staining, antigens were retrieved with 0.01 M citrate buffer (pH 6.0) for 30 min in a water bath. The endogenous peroxidase was deactivated with 3% H_2_O_2_ in methanol for 10 min. After blocking with 5% BSA for 15 min at room temperature, the sections were incubated with primary antibody (1:200 in diluting buffer, PBS, 0.01%[v/v] Triton X-100, 0.01%[v/v] Tween 20 and 5% BSA) at 4 °C overnight, followed by 30-min incubation with secondary antibody. After rinsing, the sections were incubated with DAB reagent for 1–5 min until the desired color developed. Finally, the slides were dehydrated and mounted.

### Statistical analysis

In vitro data were presented as mean ± SEM from three independent experiments. Micro-CT data were presented as mean ± SD. Statistical significance was assessed by two-tailed unpaired Student’s t test for comparison between two groups through GraphPad Prism 9. For comparison of multiple groups of data with two independent variables, two-way ANOVA followed by Bonferroni posttest was used. *P* < 0.05 was regarded as statistically significant.

## Results

### Depletion of TM9SF4 promotes osteogenic differentiation of MSCs

**Fig. 1:**
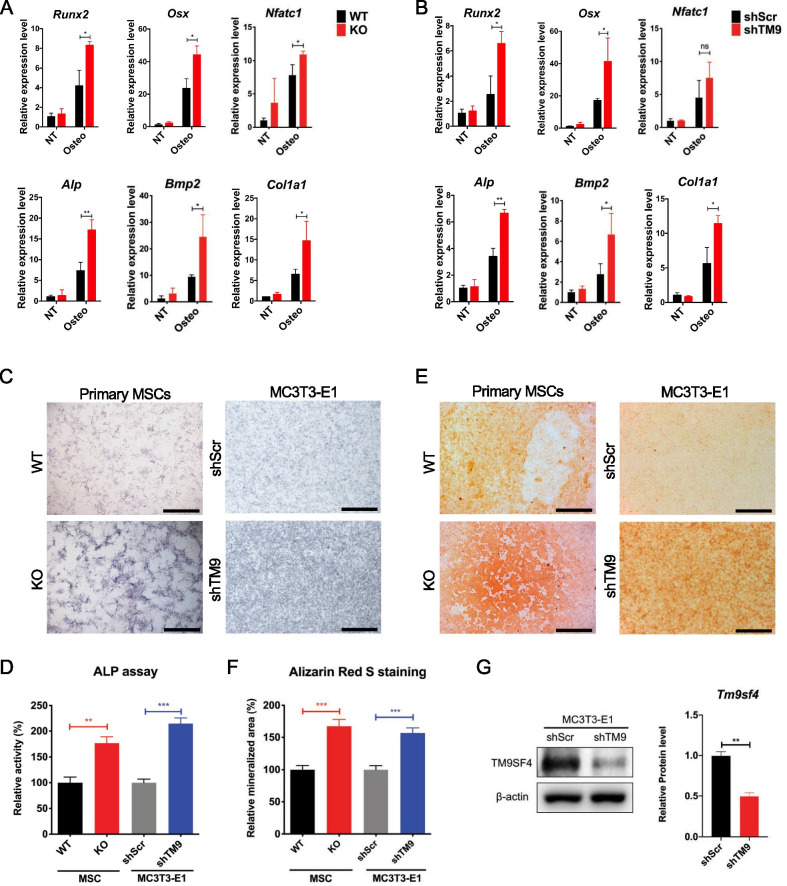
TM9SF4 deficiency promotes osteogenesis. **A**, **B** Primary MSCs and MC3T3-E1 cell lines were cultured in osteogenic medium for 7d and 5d, respectively, before qRT-PCR detection. Error bars represent mean ± SEM, n = 3 independent experiments. *P < 0.05, **P < 0.01, ns = not significant. **C**, **D** Primary MSCs were induced in osteogenic medium for 2w and MC3T3-E1 cell lines for 5d before ALP staining. Scale bars = 1 mm. **E**, **F** Calcium deposition was tested via Alizarin Red S staining after osteogenic induction, primary MSCs for 2w, MC3T3-E1 cell lines for 10d. Scale bars = 1 mm. **G** Knockdown efficiency of TM9SF4 in MC3T3-E1 cells was detected via Western blot. Error bars represent mean ± SEM, n = 3. **P < 0.01, ***P < 0.001 (**D**, **F**, **G**)

The role of TM9SF4 proteins in osteogenic differentiation of MSCs in vitro were studied first. TM9SF4 was depleted either by genetic knockout (TM9SF4^−/−^) in primary MSCs or by lentiviral shRNA-mediated stable knockdown (shTM9) in a pre-osteoblast cell line MC3T3-E1. Primary MSCs, derived from the bone marrow of TM9SF4^+/+^ and TM9SF4^−/−^ mice, were characterized phenotypically (Additional file 1: Fig. S1). These cells were treated with osteogenic medium to induce osteogenesis. The results clearly showed that genetic knockout of TM9SF4 elevated the expression levels of most osteogenic makers (Fig. 1A), stimulated the activity of alkaline phosphatase (ALP) (Fig. 1C, D), and increased the calcium deposition as detected by Alizarin Red S staining (Fig. 1E, F) in differentiated osteoblasts. Similarly, shRNA-mediated knockdown of TM9SF4 (Fig. 1G) in MC3T3-E1 cells also elevated the expression levels of most osteogenic makers (Fig. 1B) and the activity of ALP (Fig. 1C, D), as well as the deposition of calcium minerals (Fig. 1E, F) during osteogenic differentiation.

Bone remodeling is the balanced outcome of both bone formation by osteoblasts and bone resorption by osteoclasts. Therefore, we also examined the role of TM9SF4 in osteoclastogenesis. Here, bone marrow-derived monocytes were firstly used to differentiate into macrophages, which were further induced into osteoclasts. The results showed that genetic knockout of TM9SF4 did not alter the expression of osteoclastic markers, including *Acp5* (encoding tartrate-resistant acid phosphatase, TRAP)*, Tnfrsf11b* (encoding osteoprotegerin, OPG)*, Src* (encoding c-Src kinase) *and Ctsk* (encoding cathepsin K, CatK) (Additional file 2: Fig. S2A). TRAP is an acid phosphatase in osteoclasts responsible for dissolving bone matrix. TRAP staining and TRAP activity assay showed that genetic knockout of TM9SF4 had no significant effect on TRAP level/activity in differentiated osteoclasts (Additional file 2: Fig. S2B, C).

Collectively, these results strongly suggest that TM9SF4 depletion promotes the osteogenic differentiation of MSCs in vitro.

### Knockout of TM9SF4 inhibits adipogenic differentiation of MSCs

**Fig. 2:**
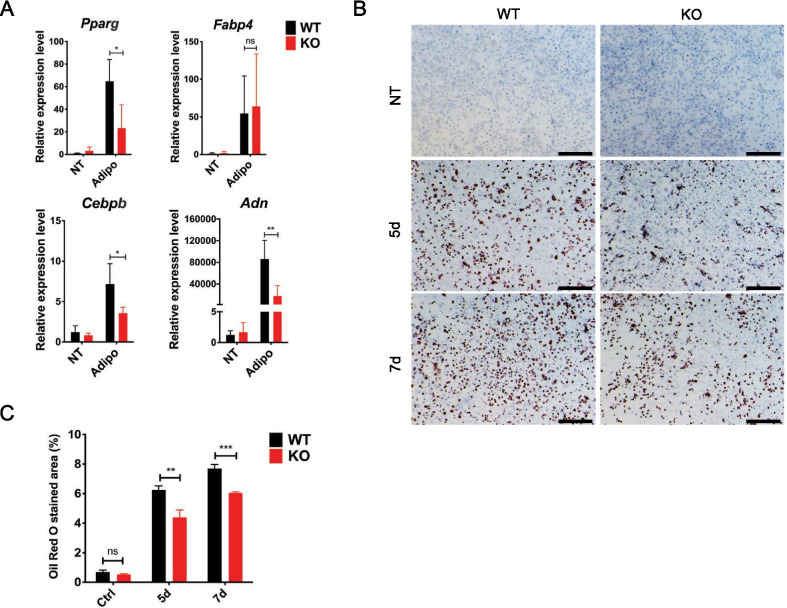
TM9SF4 knockout inhibits adipogenic differentiation of primary MSCs. **A** Primary MSCs were cultured in adipogenic medium for 5d before detection by qRT-PCR. Error bars represent mean ± SEM, n = 3, *P < 0.05, **P < 0.01, ns = not significant. **B**, **C** After adipogenic induction for 5d or 7d, the cells were fixed for Oil Red O staining. Scale bars = 500 µm. Error bars represent mean ± SEM, n = 3. **P < 0.01, ***P < 0.001, ns = not significant

Primary TM9SF4^+/+^ and TM9SF4^−/−^ MSCs were treated with adipogenic medium to induce adipogenic differentiation. The results showed that genetic knockout of TM9SF4 reduced the expression of most adipogenic genes, including *Pparg* (encoding peroxisome proliferator activated receptor gamma, PPARγ), *Cebpb* (encoding CCAAT-enhancer-binding protein, C/EBPβ), *Adn* (encoding adipsin), *Adpn* (encoding adiponectin) and *Fabp4* (encoding fatty acid binding protein 4, αFABP) (Fig. 2A), and decreased lipid accumulation as determined by Oil Red O staining (Fig. 2B, C) in differentiated adipocytes. Based on the above results, TM9SF4 is speculated to promote osteogenesis and inhibit adipogenesis, without affecting osteoclastogenesis in vitro.

### TM9SF4 knockout reduces bone loss in ovariectomy-induced osteoporotic mice

**Fig. 3:**
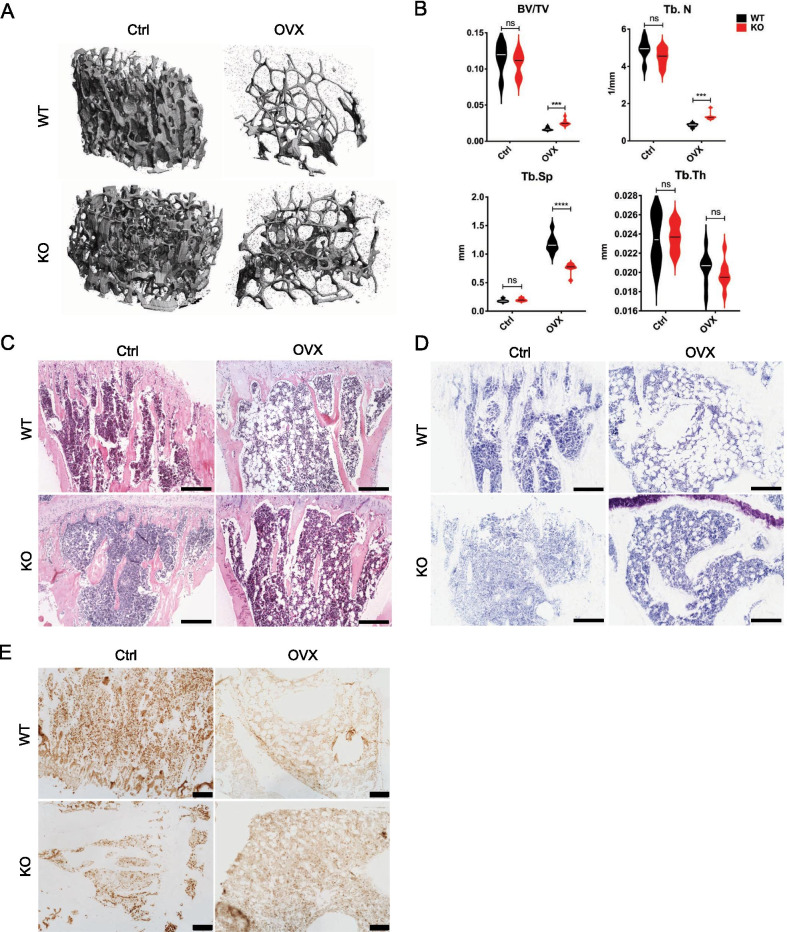
TM9SF4 depletion delays bone loss during osteoporosis. **A**, **B** Micro-CT analysis of trabecular structure in femurs from normal adult mice and OVX mice. Ctrl, non-surgery group; OVX, ovariectomy group. BV/TV, bone volume/total volume; Tb.N, trabecular number; Tb.Sp, trabecular spacing; Tb.Th, trabecular thickness. Error bars represent mean ± SD, n = 6 in Ctrl group, n = 8 in OVX group, ***P < 0.001, ****P < 0.0001, ns = not significant. **C**, **D** H&E staining and toluidine blue staining on the top of tibias. Scale bars = 150 µm. **E** IHC staining of Collagen1α1. Scale bars = 150 µm

The role of TM9SF4 in osteoporosis in vivo was further examined using an ovariectomy-induced osteoporotic mouse model. Three months after ovariectomy, bone density of TM9SF4^+/+^ and TM9SF4^−/−^ mice was measured through micro-CT (Fig. 3A, B). Notably, TM9SF4^−/−^ mice showed relative higher bone density (BV/TV) than TM9SF4^+/+^ mice (Fig. 3A, B). Also, more trabecular bones were remained in TM9SF4^−/−^ mice, as indicated by increased trabecular number and less trabecular spacing (Fig. 3A, B). However, the trabecular thickness was not significantly changed by TM9SF4 knockout (Fig. 3B). The H&E staining and toluidine blue staining showed more vacuoles formed in TM9SF4^+/+^ groups, suggesting more adipocytes and lipid accumulation in the bone marrow of TM9SF4^+/+^ mice (Fig. 3C, D). Besides, there was more Collagen1α1 (Col1a1), which is the main component in bone matrix, shown in the TM9SF4^−/−^ mice-derived tissue sections than the corresponding TM9SF4^+/+^ group from IHC staining (Fig. 3E), which is consistent with the in vitro results (Fig. 1E).

### Wnt/β-catenin pathway is essential for TM9SF4 depletion-induced osteogenic differentiation

**Fig. 4:**
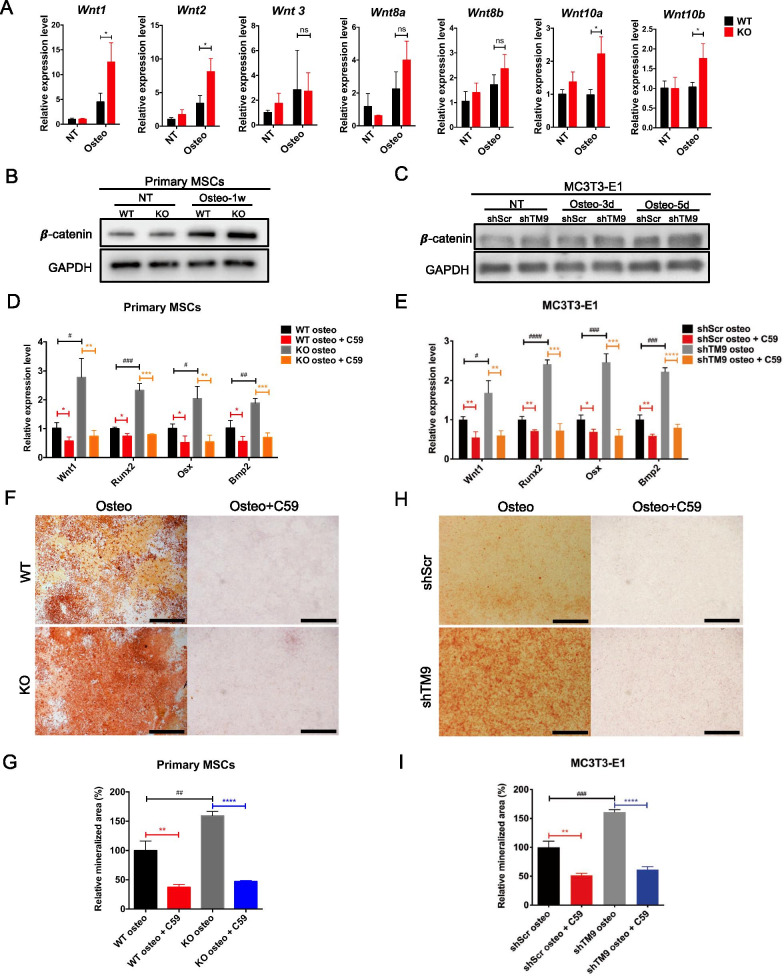
Canonical Wnt pathway is involved in TM9SF4-regulated osteogenesis. **A** Wnt ligands were examined by qRT-PCR after primary MSCs were cultured in osteogenic medium for 5d. Error bars represent mean ± SEM, n = 3. *P < 0.05, ns = not significant. **B**, **C** Primary MSCs were induced with osteogenic medium for 1w and MC3T3-E1 cells for 3d and 5d, respectively, before Western Blot detection. **D**, **E** Primary MSCs were incubated in osteogenic medium with 10 µM Wnt-C59 for 1w and MC3T3-E1 cells for 5d before RNA detection. Error bars represent mean ± SEM, n = 3. **P < 0.01, ***P < 0.001, ****P < 0.0001. **F**, **H** Cells were induced with osteogenic medium with 10 µM Wnt-C59, primary MSCs for 2w and MC3T3-E1 cells for 10d. Scale bars = 1 mm. Error bars represent mean ± SEM, n = 3. **P < 0.01, ***P < 0.001, ****P < 0.0001 (**G**, **I**)

Due to the great importance of canonical Wnt pathway during osteogenesis, next we investigated the involvement of Wnt/β-catenin in TM9SF4 depletion-induced osteogenic differentiation. Firstly, the osteoblasts differentiated from TM9SF4^−/−^ MSCs showed overall higher mRNA levels of most Wnt ligands, indicating an overactivated state of Wnt pathway during osteogenic differentiation of MSCs after TM9SF4 depletion (Fig. 4A). Secondly, depletion of TM9SF4 either by genetic knockout or by shRNA-mediated gene silencing each increased the level of β-catenin in differentiated osteoblasts (Fig. 4B, C).

Wnt-C59, an inhibitor that suppresses the production of Wnt ligands, was used to pharmacologically block Wnt pathway during osteogenesis. Wnt-C59 treatment decreased the expression level of osteogenic markers with or without TM9SF4 depletion (Fig. 4D, E). Also, Wnt-C59 dramatically reduced calcium deposition during osteogenic differentiation of primary MSCs (Fig. 4F, G) and MC3T3-E1 cells (Fig. 4H, I) with or without TM9SF4 depletion. The above data not only prove that Wnt/β-catenin participates in TM9SF4 depletion-induced osteogenesis, but also highlights the importance of canonical Wnt pathway during osteogenic differentiation of MSCs.

### Involvement of actin polymerization in TM9SF4 depletion-induced osteogenic differentiation

**Fig. 5:**
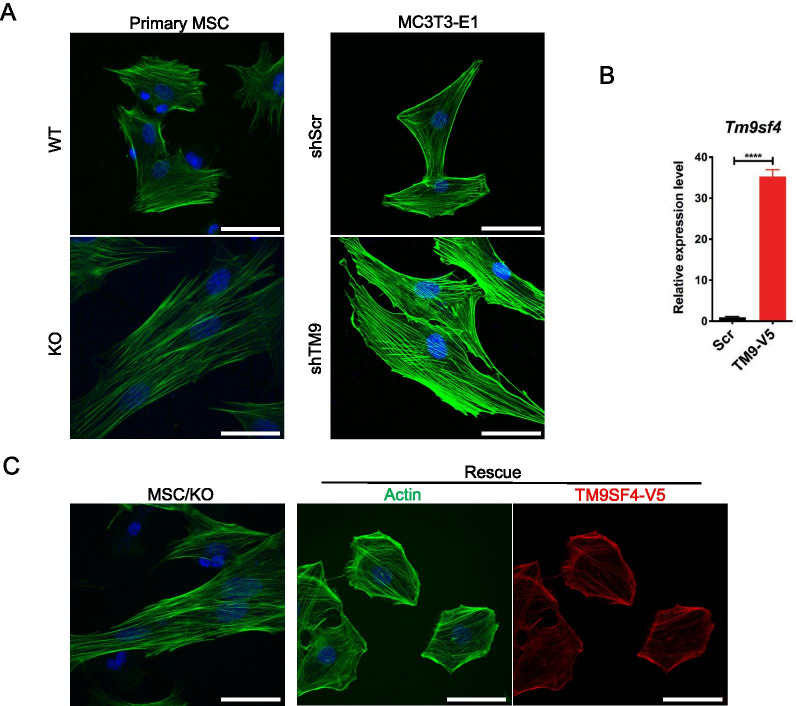
TM9SF4 suppression promotes actin filament polymerization. Actin fibers were stained with Alexa Fluor 488-phalloidin and observed under confocal microscope with 63 × oil immersion objective lens. **A** Confocal images of F-actin in primary MSCs and stable MC3T3-E1 cell lines. Scale bars = 50 µm. **B** Transfection efficiency was detected via qRT-PCR in TM9SF4^−/−^ MSCs after rescue with pcDNA6-TM9SF4-V5. Error bars represent mean ± SEM, n = 3. ****P < 0.0001*.* **C** Representative images of F-actin (green) and TM9SF4-V5 (red) in rescued MSCs. Scale bars = 50 µm

Based on the great importance of stress fibers on MSC lineage commitment [12–15], as well as the role of TM9SF4 in cytoskeleton remodeling [21], we next explored the possible involvement of F-actin polymerization in TM9SF4 depletion-induced osteogenic differentiation. We found that TM9SF4 depleted MSCs or MC3T3-E1 cells displayed elongated cell shape with longer and denser stress fibers than those in control MSCs (Fig. 5A). Overexpression of TM9SF4 in TM9SF4^−/−^ MSCs could rescue this phenotype, leading to smaller cell size with thinner and shorter actin fibers (Fig. 5B, C).

**Fig. 6:**
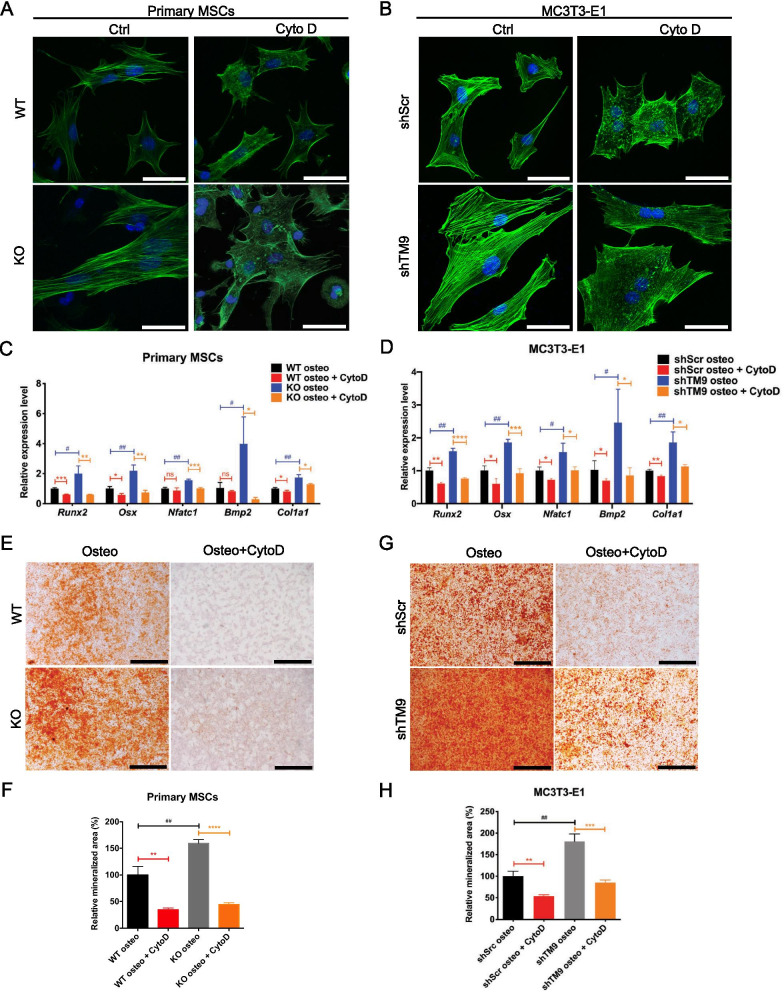
Cytochalasin D suppresses osteogenesis by disrupting actin fibers. **A**, **B** Confocal images of actin fibers in primary MSCs and ME3T3-E1 cell lines after treated with 0.1 µg/ml CytoD for 24 h. Scale bars = 50 µm. **C**, **D** Osteogenic markers were detected by qRT-PCR after induced with osteogenic medium, primary MSCs for 1w, MC3T3-E1 cells for 5d. Error bars represent mean ± SEM, n = 3. *P < 0.05, **P < 0.01, ***P < 0.001, ****P < 0.0001, ns = not significant. **E**–**H** Alizarin Red S staining after osteogenesis, primary MSCs for 2w, MC3T3-E1 cells for 10d. Scale bars = 1 mm. Error bars represent mean ± SEM, n = 3. *P < 0.05, **P < 0.01, ***P < 0.001, ****P < 0.0001, ns = not significant (**F**, **H**)

Cytochalasin D (CytoD) was used to disrupt actin polymerization in cells. Confocal microscope pictures confirmed that 0.1 μg/mL CytoD was able to effectively induce actin depolymerization after 24 h in both primary MSCs and MC3T3-E1 cells (Fig. 6A, B). Interestingly, CytoD treatment also decreased the expression of osteogenic markers during the osteogenic differentiation of primary MSCs and MC3T3-E1 cells (Fig. 6C, D). In Alizarin Red S staining, CytoD treatment dramatically reduced mineral deposition during osteogenesis (Fig. 6E–H). The impediment of osteogenesis by CytoD existed in both control and TM9SF4-depleted groups, indicating that the integrity of actin fibers was related to osteogenesis. This effect was even more significant in TM9SF4-depleted cells, providing supporting evidence that TM9SF4 depletion promotes the osteogenic differentiation of MSCs partially through strengthening the actin fiber integrity.

### mTORC2/Akt is involved in TM9SF4 depletion-induced osteogenic differentiation

**Fig. 7:**
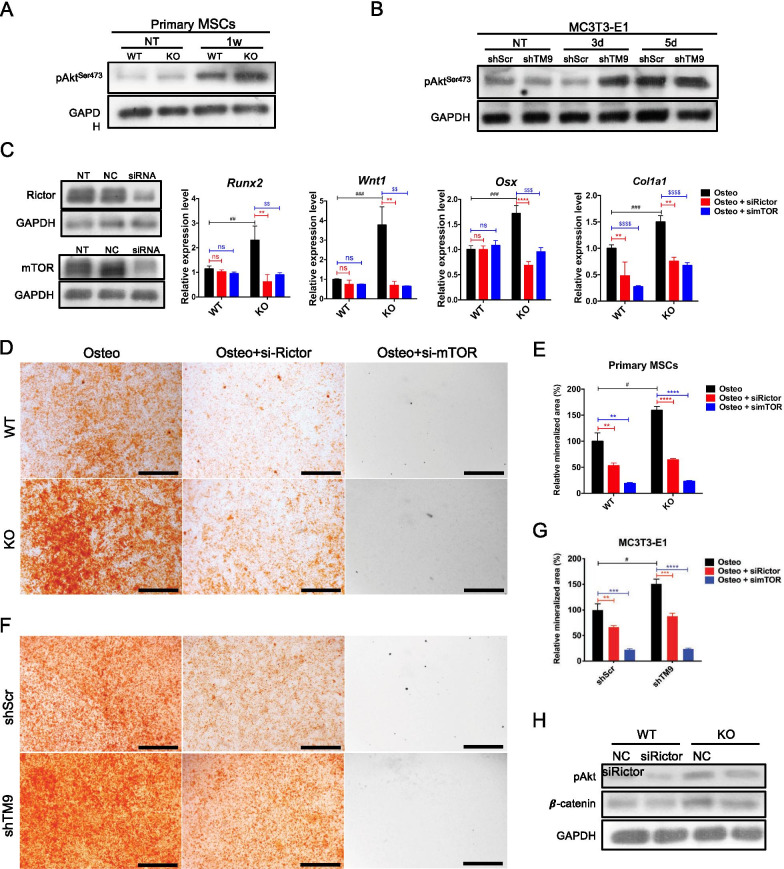
mTORC2 is necessary for TM9SF4-mediated osteogenesis. **A**, **B** The protein level of pAkt was detected via Western blot after osteogenic induction. **C** Primary MSCs with siRNA transfection were cultured in osteogenic medium for 1w before RNA detection. Error bars represent mean ± SEM, n = 3. **P < 0.01, ***P < 0.001, ****P < 0.0001, ns = not significant. **D**–**G** After siRNA transfection, primary MSCs were induced with osteogenic medium for 2w and MC3T3-E1 cells for 10d before Alizarin Red S staining. Scale bars = 500 µm. Error bars represent mean ± SEM, n = 3. **P < 0.01, ***P < 0.001, ****P < 0.0001 (**E**, **G**). **H** After siRNA transfection, primary MSCs were cultured in osteogenic medium for 1w before Western blot detection

The involvement of stress fibers in MSC differentiation relies on intracellular mechanosensitive signaling pathways. It has been reported that mammalian target of rapamycin complex 2 (mTORC2) and its downstream target Akt could regulate MSC lineage selection through cytoskeletal remodeling. Therefore, the role of mTORC2 and Akt in TM9SF4 depletion-induced osteogenic differentiation was also investigated. The Western blot results showed that Akt phosphorylation at Ser473 was upregulated in TM9SF4-depleted cells during osteogenic differentiation (Fig. 7A, B). siRNA-mediated knockdown of either Rictor, the key subunit in mTORC2, or mTOR could decrease the expression superiority of osteogenic markers in TM9SF4^−/−^ MSCs (Fig. 7C). At the same time, knockdown of Rictor or mTOR also reduced the TM9SF4 depletion-induced calcium deposition during osteogenic differentiation of primary MSCs (Fig. 7D, E) and MC3T3-E1 cells (Fig. 7F, G).

We next confirmed that mTORC2 exerted the pro-osteogenic action through the phosphorylation of Akt at Ser473. Knockdown of Rictor reversed the TM9SF4 depletion-induced upregulation of pAkt^Ser473^ and β-catenin (Fig. 7H), supporting that pAkt^Ser473^, situated downstream of mTORC2, indeed participated in TM9SF4 depletion-induced osteogenesis. The above data suggest that TM9SF4 depletion promotes osteogenic differentiation of MSCs through a mTORC2/Akt/β-catenin cascade.

## Discussion

Lineage commitment of MSCs into osteoblasts or adipocytes is competitive and reciprocal [3]. A variety of chemical and physical factors, such as growth hormones and cytoskeletal reorganization, can regulate the delicate balance of MSC differentiation into osteoblasts or adipocytes. However, the detailed mechanism of such regulation is not well understood. In the present study, we explored the possible role of TM9SF4 in the regulation of MSC lineage commitment. Depletion of TM9SF4, either by gene knockout in primary MSCs or by shRNA-mediated knockdown in a pre-osteoblast cell line MC3T3-E1, promoted osteogenic ability, as indicated by elevated expression of multiple osteogenic genes, enhanced ALP activity and increased calcium deposition. In addition, TM9SF4 depletion reduced adipogenic ability, as indicated by decreased expression of adipogenic genes and less lipid accumulation. Together, our studies demonstrate TM9SF4 as a novel protein that regulates the switch of MSC differentiation between osteoblasts and adipocytes. Specifically, TM9SF4 deficiency has an apparent pro-osteogenic and anti-adipogenic effect in vitro.

Delicate balance of MSC differentiation to osteoblasts or adipocytes is important in the maintenance of bone homeostasis, while its dysregulation may lead to osteoporosis, which is a major health problem in aging people [2, 5]. Therefore, we next explored the role of TM9SF4 in osteoporosis in vivo using ovariectomized mice. The results showed that ovariectomy-induced osteoporosis could be partially reversed by TM9SF4 knockout, as indicated by multiple bone mass-related parameters in micro-CT, including BV/TV, Tb.N and Tb.Sp. Osteoblast staining of bone section demonstrated that the trabecular bones from TM9SF4^−/−^ mice contained more bone matrix but less adipocytes than those from TM9SF4^+/+^ mice. These data demonstrate that TM9SF4 knockout has pro-osteogenic but anti-adipogenic role in osteoporotic mouse model. Previously, TM9SF4 knockout mice were known to have abnormal thoracic cage shape and vertebrate transformation, suggesting a possible role of TM9SF4 in bone formation. Our present study provides the first evidence that targeting TM9SF4 could be helpful for the prevention or treatment of osteoporosis.

The recognition of the molecular mechanisms regulating MSC lineage commitment between osteoblasts and adipocytes is important for understanding the pathological mechanism of osteoporosis. Next, we explored the signaling pathways downstream of TM9SF4 in the osteo-adipogenic lineage competition. Wnt/β-catenin, BMP and Notch pathways are known to play key roles in osteoblast and adipocyte differentiation of MSCs. Among them, Wnt/β-catenin has a clearly opposite effect on osteogenic and adipogenic differentiation of MSCs, being pro-osteogenic but anti-adipogenic [2]. Therefore, we focused on the Wnt/β-catenin pathway in this study. The results showed that osteogenic differentiation increased the expression levels of most Wnt ligands and β-catenin, which were further elevated by TM9SF4 knockout/knockdown. Importantly, inhibition of Wnt pathway by Wnt-C59 abolished the TM9SF4 depletion-induced osteogenic differentiation. These results strongly support that TM9SF4 depletion acts through Wnt/β-catenin pathway to promote osteogenic differentiation of MSCs.

Cell shape, F-actin dynamics and cytoskeletal organization are among the physical factors that can regulate the balance of MSC lineage commitment between osteoblasts and adipocytes [3, 16, 17]. It is known that F-actin polymerization promotes MSC differentiation to osteoblasts, whereas F-actin depolymerization favors MSC commitment to adipocytes [12–14]. TM9SF4 was previously reported to alter cell shape and actin reorganization in *Drosophila* macrophages [21]. Therefore, we explored whether TM9SF4 could affect cell shape and F-actin organization in MSCs, thereafter impacting their lineage commitment. Indeed, we found that depletion of TM9SF4 in primary MSCs and pre-osteoblast MC3T3-E1 cells resulted in cell elongation with longer and denser F-actin stress fibers. Furthermore, disruption of F-actin polymerization by CytoD abolished the TM9SF4 depletion-induced osteogenic differentiation of MSCs. These results support the idea that TM9SF4 depletion might act through F-actin dynamics to promote osteogenic differentiation of MSCs.

It is well documented that F-actin polymerization may activate mTORC2/Akt/β-catenin to promote MSC osteogenic differentiation [16–18]. mTORC2 phosphorylates Akt at Ser^473^ to mediate this effect [25]. In agreement, our study showed that RNA silencing of either mTOR or Rictor, two key components of mTORC2, abolished the TM9SF4 depletion-induced osteogenic differentiation of MSCs. Moreover, TM9SF4 depletion-induced upregulation of pAkt^473^ and β-catenin could be reversed by Rictor-silencing. Together, these results prove that TM9SF4 knockdown acts through F-actin/mTORC2/Akt/β-catenin signaling cascade to promote MSC differentiation into osteoblasts.

Another point to note is that TM9SF4 is not required for osteogenic differentiation, but rather a regulator for osteogenic differentiation, as osteogenic differentiation can proceed with or without the expression of TM9SF4 (Figs. 3, 4, 7). In the presence of TM9SF4 expression, which tends to suppress the osteogenic differentiation, osteogenic differentiation can still proceed. It is likely that the TM9SF4-independent osteogenic events also go through Wnt and F-actin pathways, because treatment with Wnt-C59 or CytoD completely abolished the osteogenic differentiation, including TM9SF4-dependent and TM9SF4-independent components (Figs. 3, 4, 7).

In conclusion, our in vitro and in vivo experiments demonstrate a novel function of TM9SF4 proteins in the control of MSC lineage commitment between osteoblasts and adipocytes. Suppression of TM9SF4 promotes osteogenic differentiation but inhibits adipogenic differentiation of MSCs. In mice, TM9SF4 knockout alleviates osteoporosis development in the ovariectomized mouse model. It is likely that TM9SF4 acts through Wnt/β-catenin and mTORC2/Akt/β-catenin cascades to exert its effect on osteo-adipogenic differentiation of MSCs, as shown in the schematic diagram (Additional file 3: Fig. S3). In future, it will be interesting to explore whether TM9SF4 could be a potential target for osteoporosis treatment.

## Supplementary Information

**Additional file 1. Figure S1**. Phenotypic characterization of mouse bone marrow-derived MSCs.

**Additional file 2. Figure S2**. TM9SF4 has no impact on osteoclastogenesis.

**Additional file 3. Figure S3**. Working model of TM9SF4-regulated mTORC2/Akt/β-catenin and Wnt/β-catenin activation during bone formation.

## Data Availability

qRT-PCR data, Western blot data and all staining data generated in this study are included in this article. All other data and materials are available from the corresponding author upon reasonable request.

## Abbreviations

Adn: Adipsin
Adpn: Adiponectin
ALP: Alkaline phosphatase
BMP: Bone morphogenetic protein
BV/TV: Bone volume/total volume
CatK: Cathepsin K
C/EBPβ: CCAAT-enhancer-binding protein
Col1a1: Collagen1α1
CytoD: Cytochalasin D
αFABP: Fatty acid-binding protein 4
IHC: Immunohistochemistry
M-CSF: Macrophage colony-stimulating factor
micro-CT: Micro-computed tomography
mTORC2: Mammalian target of rapamycin complex 2
MSC: Mesenchymal stem cells
NFATc1: Nuclear factor of activated T Cells 1
OPG: Osteoprotegerin
OSX: Osterix
PPARγ: Peroxisome proliferator-activated receptor gamma
RANKL: Receptor activator for nuclear factor-κB Ligand
Rictor: Rapamycin-insensitive companion of mTOR
Runx2: Runt-related transcription factor 2
Src: c-Src kinase
Tb.N: Trabecular number
Tb.Sp: Trabecular spacing
TM9SF4: Transmembrane 9 superfamily 4
TRAP: Tartrate-resistant acid phosphatase
