# Supplementary figures and images for: TM9SF4 is a novel regulator in lineage commitment of bone marrow mesenchymal stem cells to either osteoblasts or adipocytes
Source: Stem Cell Res Ther. 2021 Nov 13;12:573. doi: 10.1186/s13287-021-02636-8 (PMC8590266; doi:10.1186/s13287-021-02636-8)

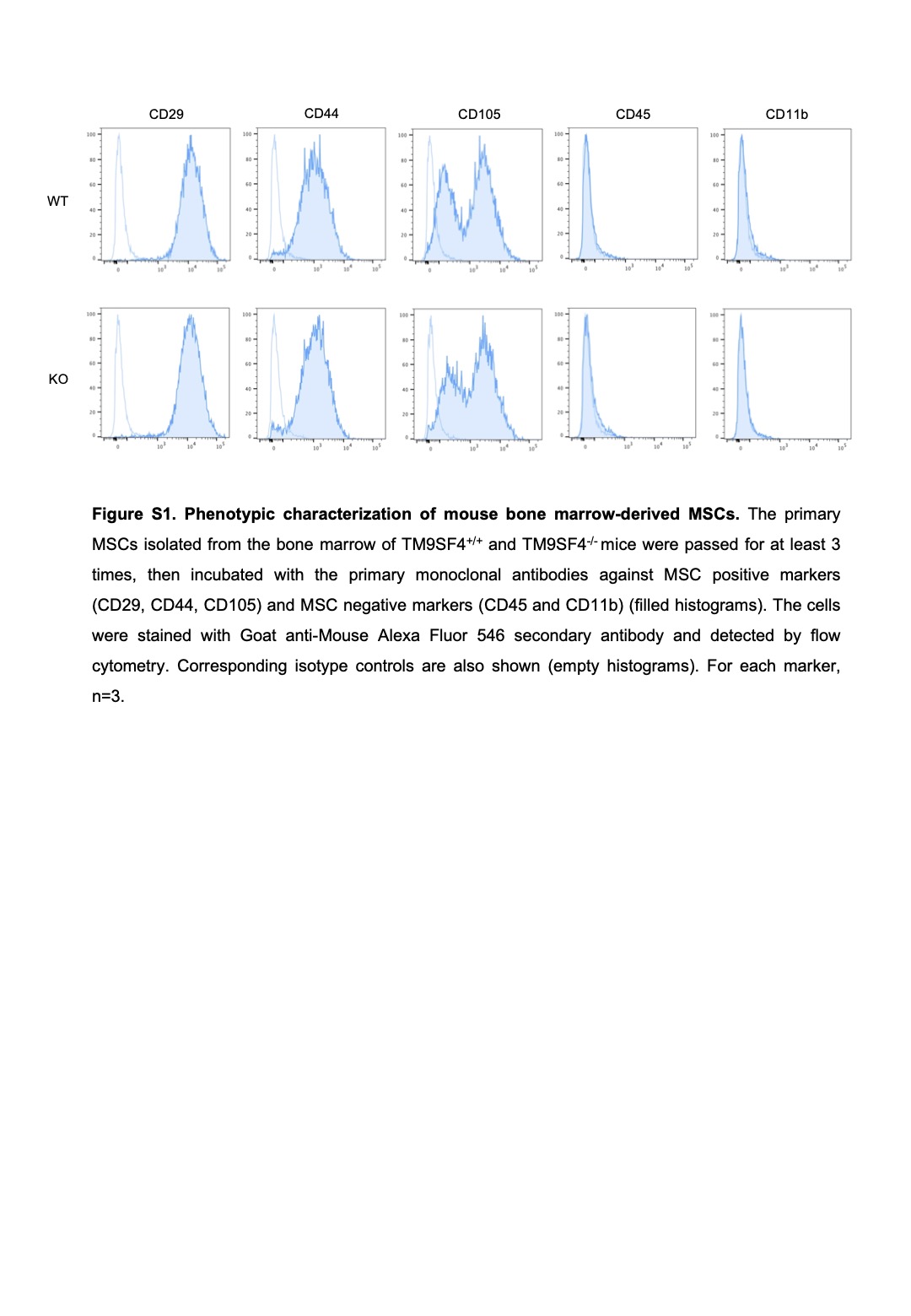

Supplement: Supplementary file 1 — Additional file 1. Figure S1. Phenotypic characterization of mouse bone marrow-derived MSCs. [file 13287_2021_2636_MOESM1_ESM.jpg]

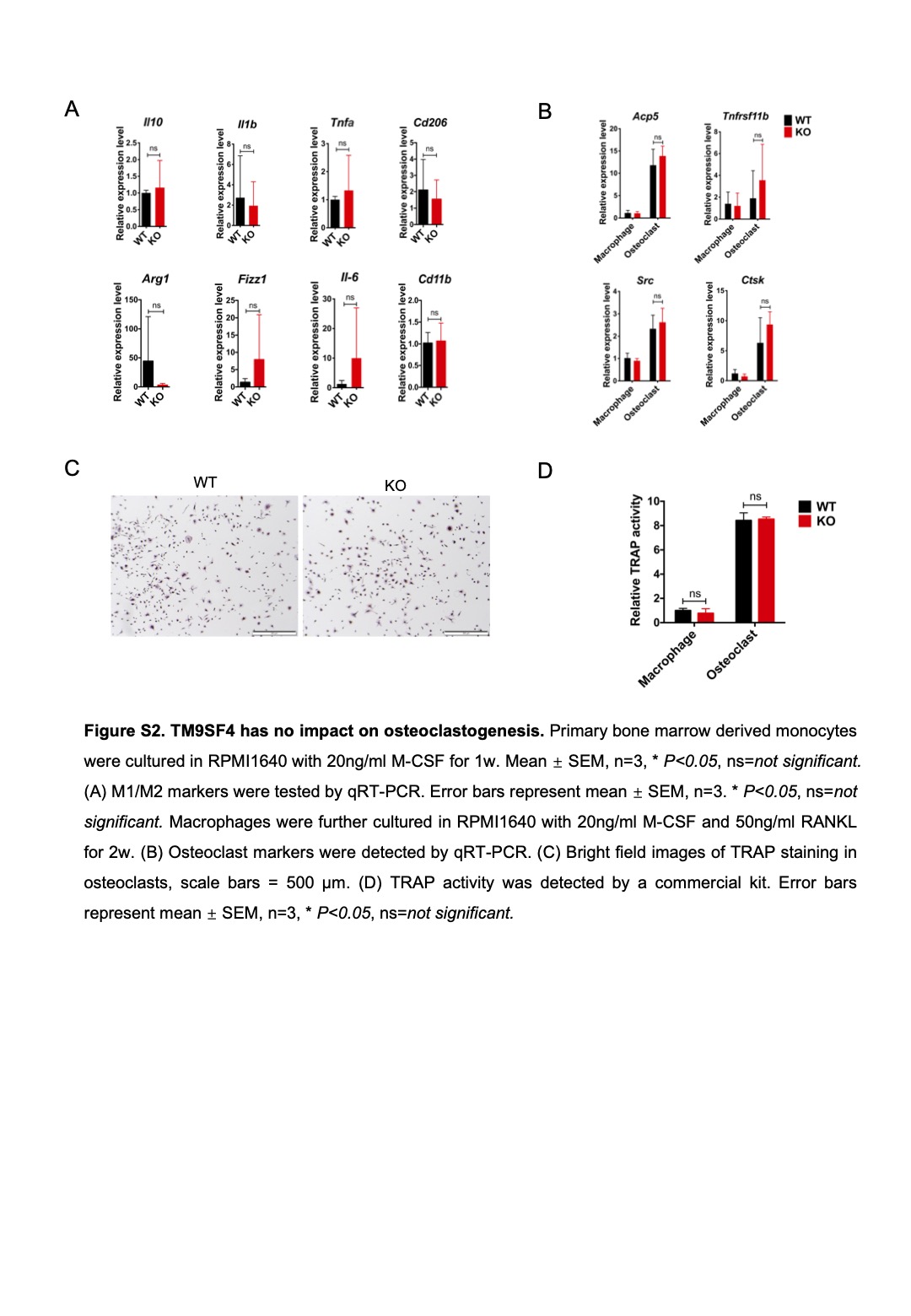

Supplement: Supplementary file 2 — Additional file 2. Figure S2. TM9SF4 has no impact on osteoclastogenesis. [file 13287_2021_2636_MOESM2_ESM.jpg]

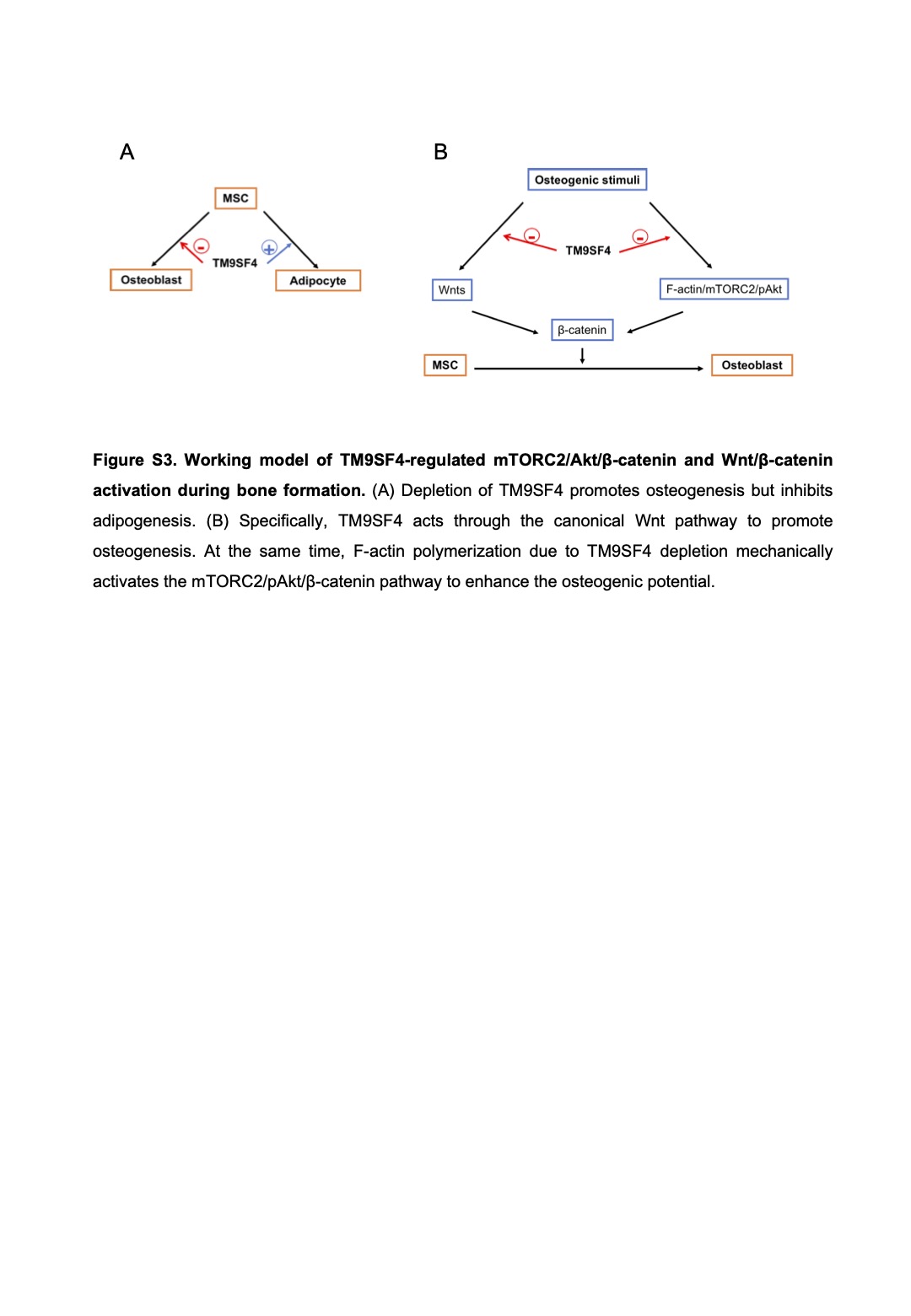

Supplement: Supplementary file 3 — Additional file 3. Figure S3. Working model of TM9SF4-regulated mTORC2/Akt/β-catenin and Wnt/β-catenin activation during bone formation. [file 13287_2021_2636_MOESM3_ESM.jpg]
